# Blood Occludin Level as a Potential Biomarker for Early Blood Brain Barrier Damage Following Ischemic Stroke

**DOI:** 10.1038/srep40331

**Published:** 2017-01-12

**Authors:** Rong Pan, Kewei Yu, Theodore Weatherwax, Handong Zheng, Wenlan Liu, Ke Jian Liu

**Affiliations:** 1 Department of Pharmaceutical Sciences, University of New Mexico Health Sciences Center, Albuquerque, NM 87131, USA; 2 Department of Rehabilitation, Huashan Hospital, Fudan University, Shanghai, 200040, China; 3 The Central Laboratory, Shenzhen Key Laboratory of Neurosurgery, Shenzhen Second People’s Hospital, Shenzhen University 1st Affiliated Hospital, Shenzhen, Guangdong, 518035, China

## Abstract

Concern about intracerebral hemorrhage (ICH) is the primary reason for withholding tPA therapy from patients with ischemic stroke. Early blood brain barrier (BBB) damage is the major risk factor for fatal post-thrombolysis ICH, but rapidly assessing BBB damage before tPA administration is highly challenging. We recently reported that ischemia induced rapid degradation of tight junction protein occludin in cerebromicrovessels. The present study investigates whether the cleaved occludin is released into the blood stream and how blood occludin levels correlate to the extent of BBB damage using a rat model of ischemic stroke. Cerebral ischemia induced a time-dependent increase of blood occludin with a sharp increase at 4.5-hour post-ischemia onset, which concurrently occurred with the loss of occludin from ischemic cerebral microvessels and a massive BBB leakage at 4.5-hour post-ischemia. Two major occludin fragments were identified in the blood during cerebral ischemia. Furthermore, blood occludin levels remained significantly higher than its basal level within the first 24 hours after ischemia onset. Our findings demonstrate that blood occludin levels correlate well with the extent of BBB damage and thus may serve as a clinically relevant biomarker for evaluating the risk of ICH before tPA administration.

Stroke is a leading cause of death and adult disability. Thrombolytic therapy with tissue plasminogen activator (tPA) remains the only FDA-approved treatment for acute ischemic stroke. However, only a small fraction of stroke patients receive tPA therapy^1^. Concern about unmanageable intracerebral hemorrhage (ICH) is the major barrier to greater utilization of tPA for acute stroke thrombolysis^2^. Evidence from randomized clinical trials and subsequent clinical experience clearly demonstrated that tPA thrombolysis is associated with a 10-fold increase of ICH. Moreover, once ICH occurs, over 80% of the patients will die^3^. Currently, FDA approval of tPA requires it be used within a 4.5-h window^4^. However, the one-size-fits-all time window locks many stroke patients with a low risk of ICH out of tPA’s benefit. Thus, there is an urgent need to seek a reliable early diagnostic indicator to exclude “eligible patients” (within the thrombolytic time window) at high risk of ICH, and to include “non-eligible patients” (beyond the 4.5-h limit, but still presenting a salvageable penumbra and with low risk of ICH) for tPA treatment, allowing more stroke patients to benefit from tPA treatment.

Blood brain barrier (BBB) disruption is a hypothesized precursor to ICH^5^. Animal and human stroke studies suggest a causal predictive relationship between early (within 4–5 hours of stroke onset) ischemic BBB damage and tPA-associated ICH^5^,^6^,^7^,^8^,^9^, in which the ischemic brain regions with compromised BBB at the time of tPA administration are found to be at high risk of hemorrhagic transformation at later times during thrombolytic reperfusion. Early ischemic BBB damage is increasingly considered as a promising pretreatment predictor for post-thrombolysis ICH^7^,^8^,^10^. However, quick and quantitative assessment of early BBB damage remains a technical challenge in ischemic stroke.

Occludin is a tight junction protein that is a key structural component of the BBB^11^. Degradation of occludin is frequently seen in ischemic stroke and contributes to BBB disruption^12^,^13^,^14^,^15^. Recently, we observed rapid loss of occludin from ischemic cerebral microvessels in a rat model of ischemic stroke^14^. However, it is unknown if the degraded or cleaved occludin is released into peripheral circulation and if blood occludin levels correlate to the degree of BBB damage in the early phase of ischemic stroke. This study was aimed to answer these important questions in a rat model of cerebral ischemia.

## Results

### BBB integrity is severely damaged after 4.5-hour MCAO

**Figure 1:**
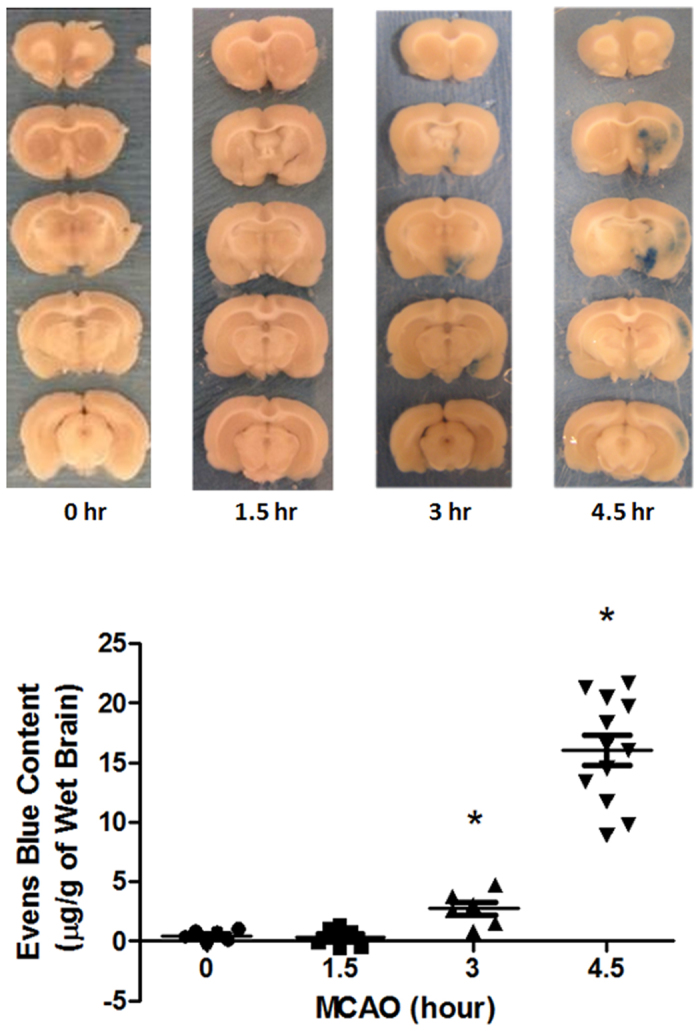
Cerebral ischemia induces BBB damage in an ischemia duration time-dependent manner. BBB permeability was assessed by measuring Evans blue leakage after indicated MCAO durations. Data were presented as mean ± SEM (0, 1.5, and 3 h, n = 6; 4.5 h, n = 12). *P < 0.05 versus 0-hour MCAO.

The effects of various durations of cerebral ischemia on BBB integrity were assessed by examining Evans blue dye leakage. 1.5-hour MCAO resulted in minimal Evans blue dye leakage, but was readily detectable after 3-hour MCAO with dye leakage mainly located in the ventromedial striatum (Fig. 1). Notably when MCAO was prolonged to 4.5 hours, Evans blue leakage was drastically increased, with the leakage expanding to all MCA supplied regions, including the cortex. These results indicate that cerebral ischemia induced BBB damage in an ischemia duration time-dependent manner and there seemed to be a threshold of ischemic duration that led to massive BBB damage.

### Blood occludin level is markedly increased at 4.5-hour post MCAO

**Figure 2:**
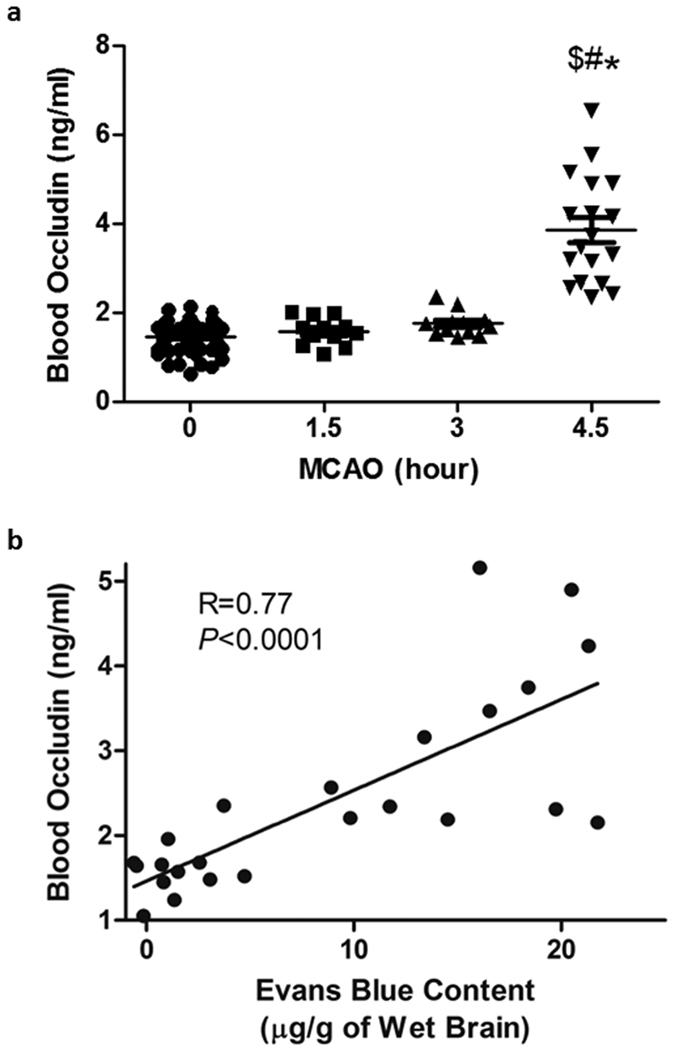
Blood occludin levels correlate to the extent of BBB damage. (**a)** ELISA was used to measure blood occludin levels after the indicated MCAO durations. Data were presented as mean ± SEM (0 h, n = 42; 1.5, and 3 h, n = 12; 4.5 h, n = 18). ^$^*P* < 0.05 versus 0-hour MCAO; *P < 0.05 versus 1.5-hour MCAO; ^#^*P* < 0.05 versus 3-hour MCAO. **(b)** Correlation between blood occludin level and BBB damage. BBB damage was quantitatively assessed by measuring Evans blue leakage (used the same data of 1.5, 3 and 4.5 h groups in Fig. 1). Linear regression analysis demonstrated a significant correlation between Evans blue leakage and blood occludin levels; *P* < 0.0001 (Pearson correlation test).

Blood occludin protein levels were assessed before MCAO onset and after indicated MCAO durations using ELISA. As shown in Fig. 2a, a low basal occludin level was detected in the blood before MCAO onset, and no significant increase was observed within the first 3 hours of MCAO. However, 4.5-hour MCAO induced a sharp increase in blood occludin level. These results suggest that release of occludin may occur after reaching certain threshold of BBB damage.

### Blood occludin level correlates to the severity of ischemic BBB damage

Next, we examined whether blood occludin level correlated to the severity of BBB damage. Indeed, a positive correlation was observed for Evans blue dye leakage and blood occludin levels within the first 4.5 hours after MCAO onset (Fig. 2b. R = 0.77, *P* < 0.05).

**Figure 3:**
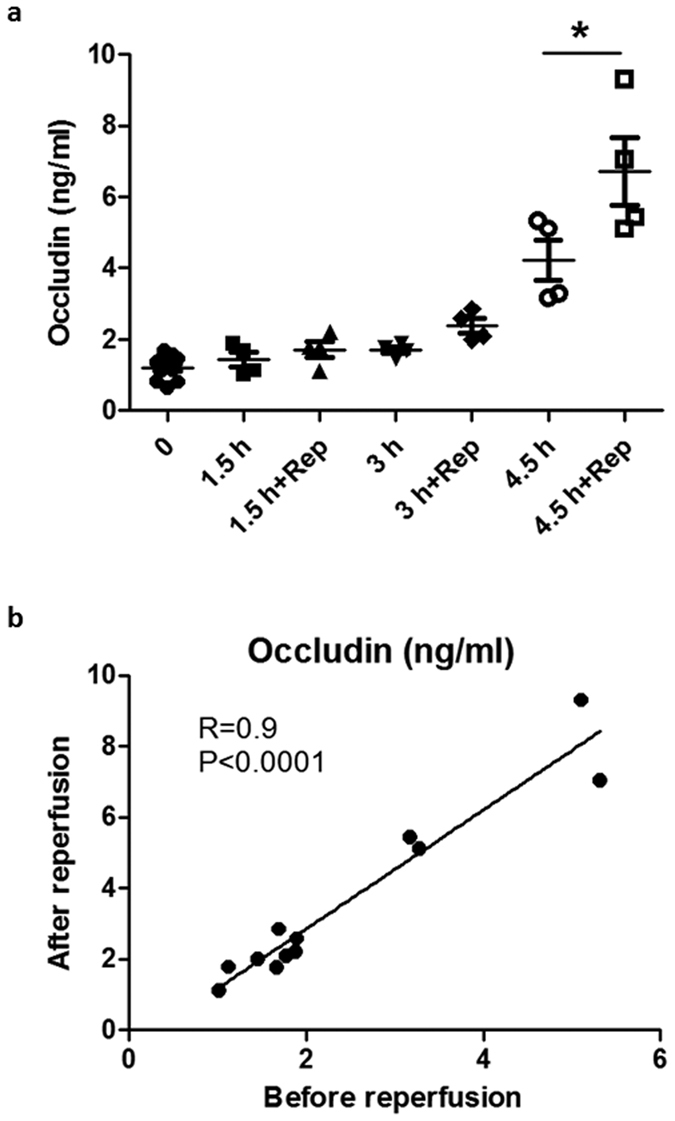
Blood occludin levels in MCAO rats without reperfusion correlate well with that with 5-min reperfusion. (**a)** Blood occludin levels at the end of the indicated MCAO time and after 5-min reperfusion, as measured by ELISA. Data were presented as mean ± SEM (0 h, n = 12; 1.5, 3 and 4.5 h, n = 4). *P < 0.05 versus without reperfusion. **(b)** Linear regression analysis demonstrated a significant correlation between the blood occludin level before and after 5-min reperfusion; *P* < 0.0001 (Pearson correlation test).

Considering that filament occlusion may not allow significant blood flow to the ischemic brain, particularly the ischemic core, which could limit the efflux of cleaved occludin from the ischemic core. In this context, most of the occludin detected above should be derived from the ischemic penumbra as blood was taken during MCAO (without reperfusion). However, our BBB damage assessment included dye leakage at the ischemic core since the measurement was done at 5 min after reperfusion. To exclude the possibility that blood occludin level assessed before reperfusion (released from the penumbra) might not well represent total release of occludin from the ischemic region (penumbra + core), we conducted another set of experiments to compare blood occludin levels assessed before reperfusion onset and 5 min after reperfusion. Figure 3a,b showed a good correlation (R = 0.9, *P* < 0.05) between blood occludin levels assessed before and after 5-min reperfusion, indicating that blood occludin level measured at the end of MCAO (without reperfusion) could well reflect occludin release from the entire ischemic brain.

### Occludin protein loss is seen in ischemic cerebral microvessels

**Figure 4:**
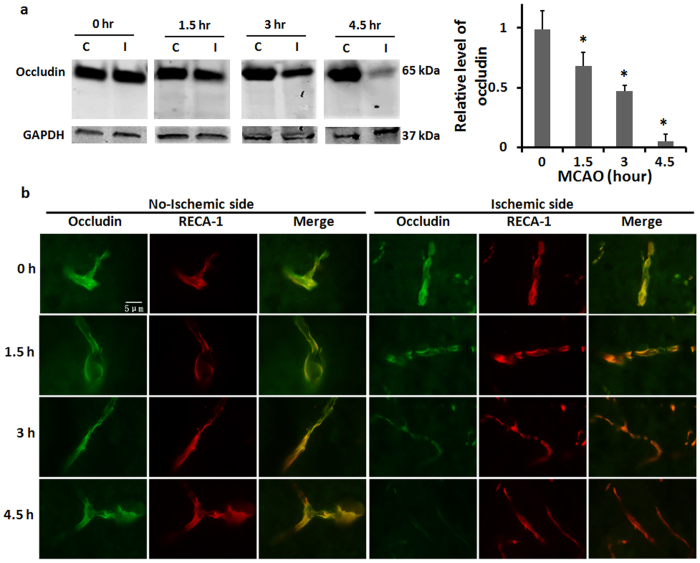
Cerebral ischemia induces rapid occludin loss from microvessels in ischemic brain. **(a)** Cerebral microvessels were isolated from nonischemic (C) and ischemic (I) hemispheric tissue after the indicated duration of MCAO and occludin protein levels in the microvessels were measured by western blot. Data were presented as mean SEM (n = 4). *P < 0.05 versus 0-hour MCAO. **(b)** Immunofluorescence was performed to show loss of occludin (green) from microvessels (stained with RECA-1, red) in brain slices of ischemic rats with indicated duration of MCAO.

To determine whether increased occludin protein in blood was due to occludin released from cerebral microvasculature, we isolated the microvessels from the ischemic brain and examined changes of occludin protein levels. Our western blot results (Fig. 4a) showed that 1.5-hour MCAO led to a slight decrease in occludin protein levels in ischemic cerebromicrovessels, and when MCAO was prolonged to 3- or 4.5 hours, loss of occludin protein on the microvessels was dramatically increased. We then further examined the occludin loss from microvessels *in situ* in brain slice using immunostaining (Fig. 4b). Consistent with Western blot results, prolonged ischemia duration caused greater loss of occludin from the microvessels. These findings indicate that loss of occludin from the microvessels occurs concurrently with the appearance of occludin in the blood circulation, suggesting that released occludin from the microvessels is a source for the occludin detected in the periphery blood.

### Two occludin fragments are detected in the periphery blood of MCAO rats

**Figure 5:**
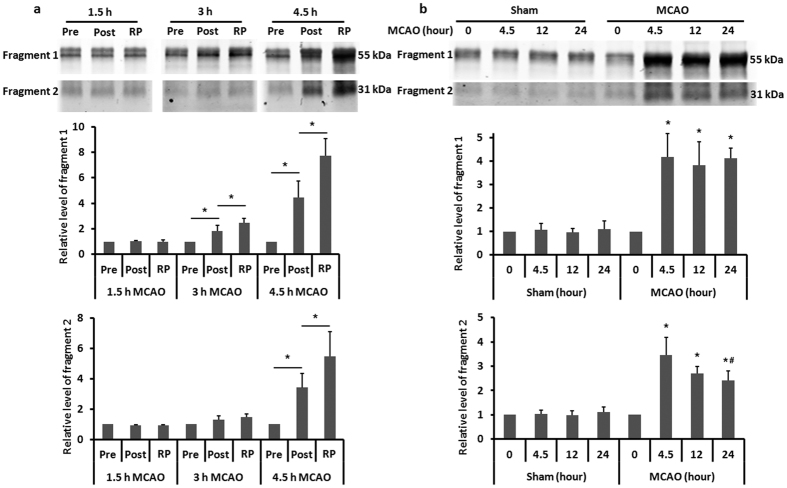
MCAO increases the levels of two cleaved occludin fragments in the periphery blood. **(a)** Immunoprecipitation plus western blot identified two major cleaved occludin fragments in the blood of MCAO rats before (Pre) and after (Post) ischemia with indicated duration, and after 5-min reperfusion (RP). Data were presented as means ± SEM (n = 3). *P < 0.05. **(b)** Blood levels of both occludin fragments were increased dramatically at 4.5-hour MCAO and remained high throughout the first 24 hours after MCAO onset. Data were presented as means ± SEM (n = 3). *P < 0.05 versus 0-hour MCAO, ^#^*P* < 0.05 versus 4.5-hour MCAO.

Occludin protein can be degraded from the cerebral microvessels by MMPs and caspases to generate 55 kDa and 31 kDa fragments, respectively^16^. To find out which occludin fragments were present in the blood samples following MCAO, we performed immunoprecipitation to pull down occludin fragments and then detected them by western blot. As shown in Fig. 5, two major occludin fragments (55 kDa and 31 kDa) were detected in the blood samples. The 55-kDa band started to increase 3-hour after MCAO, while a significant increase of the 31-kDa band was seen at the 4.5-hour MCAO time point, suggesting that the 31-kDa fragment may be a major product of severe BBB damage. Of note, although reperfusion further increased the levels of each occludin fragments, the overall trend of changes over ischemic duration was similar for rats with or without reperfusion (Fig. 5a) (compare before (Pre) vs. after (Post) ischemia data in the figure). These results indicate that cerebral ischemia generated two cleaved occludin fragments in the peripheral blood within the first 4.5 hours after ischemia onset, and that the 31-kDa fragment may be a significant biomarker for severe BBB damage.

### Blood levels of occludin fragments remain high within the first 24 hours after MCAO onset

We next investigated the change of blood occludin levels under prolonged MCAO durations (12 and 24 hours). Blood samples were collected at three time points: before MCAO onset, 12 and 24 hours after MCAO onset. As shown in Fig. 5b, the blood levels of both occludin fragments at 12- and 24-hour MCAO remained highly elevated compared to their basal levels (before MCAO onset). These findings demonstrated that the increase of blood occludin levels appears to last longer than 24 hours, suggesting that blood occludin levels may be able to reflect the extent of BBB damage even at a prolonged time period after stroke onset.

### Cerebral ischemia slightly elevates blood MMP-9 levels, but not claudin-5 levels within the first 4.5 hours of cerebral ischemia

**Figure 6:**
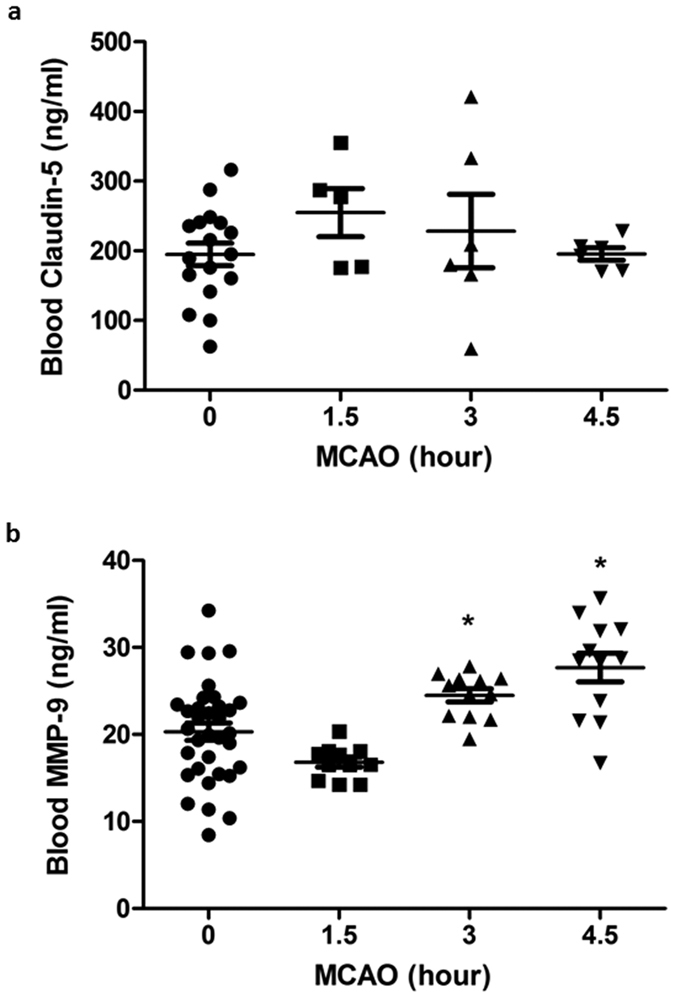
The changes of blood MMP-9 and claudin-5 within the first 4.5 hours after MCAO onset. Blood claudin-5 (**a)** (0 h, n = 17; 1.5 h, n = 5; 3 and 4.5 h, n = 6) and MMP-9 (**b)** (0 h, n = 36; 1.5, 3 and 4.5 h, n = 12) levels were measured by ELISA kits at indicated ischemic time points. Data were presented as means ± SEM. *P < 0.05 versus 0-hour MCAO.

MMP-9 and claudin-5 have been suggested as potential blood biomarkers for BBB damage and hemorrhagic transformation in stroke patients^17^,^18^. Therefore, we assessed their blood levels following MCAO. ELISA results showed that blood claudin-5 levels did not change significantly during the first 4.5 hours of cerebral ischemia (Fig. 6a), which might be due to the fact that claudin-5 protein is resistant to proteolytic degradation under stroke conditions^12^. Unlike claudin-5, blood MMP-9 levels were slightly increased at 3-hour and 4.5-hour MCAO (Fig. 6b). Of note, the basal levels of blood MMP-9 varied significantly, and the blood MMP-9 levels at 3-hour MCAO were comparable to that at 4.5-hour MCAO.

## Discussion

Despite clear benefits to ischemic stroke patients, tPA thrombolysis presents real safety concerns due to ICH^19^,^20^,^21^. Accumulating evidence supports a close association between BBB damage that occurs in the early stage of ischemic stroke and post-thrombolysis ICH^5^,^6^,^7^,^8^,^22^,^23^. Therefore, any pretreatment biomarkers associated with early ischemic BBB damage may serve as a highly helpful indicator to identify high-risk patients, thus improving the safety profile of tPA. Here we investigated the change of blood occludin levels and its association with early ischemic BBB damage. Our major findings include: 1) cerebral ischemia induces a time-dependent increase of blood occludin levels during the first 4.5 hours after MCAO onset, with a sharp increase at 4.5-hour MCAO; 2) two major occludin fragments (55 kDa and 31 kDa) are identified in the blood during cerebral ischemia; 3) BBB damage occurs early after stroke onset with a massive leakage at 4.5-hour MCAO, and blood occludin levels increase proportionately to the magnitude of BBB damage; 4) blood occludin levels remains significantly higher than its basal level within the first 24 hours after MCAO onset. These findings indicate that blood occludin may serve as a reliable early indicator for both the magnitude and duration of BBB damage in ischemic stroke patients. Since the MCAO model is well established in our lab and the focus of this study was on BBB damage and the changes of its structural protein occludin, we did not examine neurological deficits of the MCAO rats.

Since FDA’s approval of tPA thrombolysis for treating acute ischemic stroke in 1996, great efforts have been directed to discovering and developing pretreatment biomarkers for guiding stroke thrombolysis. Although a number of clinical factors (old age, hypertension, anticoagulants, hyperglycemia), radiological findings (large infarct, proximal occlusion, leukoaraiosis) and several blood biomarkers (MMP-9, c-FN, PAI-1, TAFI and S100B) have been identified and linked to an increased risk of ICH^24^,^25^,^26^, to date no reliable biomarkers are able to accurately predict the risk of ICH before tPA administration. Kazmierski *et al*. recently reported that elevated blood tight junction proteins (occludin, claudin-5 or the ratio of claudin-5/zonula occludens-1) correlate to increased incidence of clinical deterioration caused by hemorrhagic transformation in stroke patients who are not treated by tPA^17^. In this same study, occludin appears to be a better predictor of hemorrhagic transformation than claudin-5 because its levels in the circulation are close to zero in those patients without hemorrhagic transformation^17^. Thus, among the suggested blood biomarkers, blood occludin has several unique advantages for being a useful indicator of BBB damage. First, as a key structural protein sealing the BBB, the cleavage of occludin directly disrupts BBB integrity^14^,^27^,^28^. Second, occludin is highly enriched in brain capillaries, these are ~50–100 times tighter than peripheral capillaries as a result of the presence of complicated tight junctions^29^, therefore the increase in blood occludin levels mainly occurs as a result of neurovascular injury. Third, being a structural protein, occludin shows a low basal level in the circulation and a sharp increase under ischemic condition (Fig. 2), which will greatly help set a threshold value. Here, we investigated the temporal evolution of BBB damage within the first 4.5 hours of cerebral ischemia and its correlation with the change of blood occludin levels. Our data show that blood occludin levels correlates well with the extent of BBB damage at the early stroke stage, suggesting that pretreatment blood occludin levels may serve as a reliable biomarker to identify acute stroke patients at high risk of ICH. Future animal and clinical studies are warranted to test this possibility.

Consistent with previous findings that occludin degradation occurs in the ischemic brain^13^,^14^,^30^,^31^, our data also reveal a dynamic loss of occludin protein in cerebromicrovessels of the ischemic brain, with a dramatic change at 4.5-hour MCAO. Importantly, the time dependent pattern of occludin protein loss in ischemic cerebromicrovessels is concurrent with blood occludin increases in MCAO rats. This finding suggests that increased blood occludin protein levels likely result from occludin cleavage in the ischemic brain and its subsequent release into the circulation. Moreover, we obtained a good correlation (R = 0.9, *P* < 0.05) between blood occludin levels assessed before and after 5-min reperfusion (Fig. 3). It indicates that blood occludin level measured during MCAO could well reflect occludin released from the ischemic brain, which is highly related to BBB damage^14^,^27^,^28^ – the precursor of ICH after thrombolysis^5^. We further identified two cleaved occludin fragments (55 kDa and 31 kDa) in the blood of MCAO rats. Interestingly, the 55-kDa fragment started to increase at 3 hours after MCAO onset, while the 31-kDa band increased more intensely after 4.5-hour MCAO. These data indicate that blood occludin levels are increased shortly after stroke onset, with a sharp increase at 4.5 hours of cerebral ischemia. Moreover, the elevation of the 31-kDa fragment paralleled well with the increase of Evans blue leakage, suggesting that the blood levels of 31-kDa occludin fragment may reflect the extent of BBB damage. In a previous study, Bojarski *et al*. reported that occludin was degraded by MMPs and caspase-3 to generate 55 kDa and 31 kDa fragments, respectively^16^. Since caspase-3 activation is associated with apoptosis^32^, it is likely that the appearance of 31-kDa occludin fragment in the blood may mark endothelial apoptosis within the ischemic BBB, which is considered a characteristic of severe BBB damage^33^. In this context, the appearance of 31-kDa occludin fragment may indicate the beginning of serious BBB damage, which is supported by the observation of increased Evans blue dye leakage in the ischemic brain. Moreover, the increase of blood occludin levels appears to last longer than 24 hours as both 55-kDa and 31-kDa fragments remain high at 24 hours after MCAO onset. This is of critical importance for avoiding false negative results when using blood occludin to evaluate the extent of BBB damage in a clinical setting when collection of blood sample is delayed.

We also measured the changes of blood claudin-5 and MMP-9 within the first 4.5 hours after MCAO onset. Blood claudin-5 didn’t change in the early phase of cerebral ischemia while blood MMP-9 appeared to increase only marginally with prolongation of MCAO duration, suggesting the changes of these two molecules do not correlate well with the extent of early ischemic BBB damage.

In summary, our findings demonstrate that blood occludin levels correlate well to ischemic BBB damage in the early stages of ischemic strokes, suggesting that blood occludin levels could be a potential biomarker for assessing risk of ICH following tPA thrombolysis.

## Methods

### Rat model of middle cerebral artery occlusion

The Laboratory Animal Care and Use Committee of the University of New Mexico approved all experimental protocols. The animals were used in compliance with the NIH Guide for Care and Use of Laboratory Animals. We did the experiments according to the ARRIVE guidelines. Male Sprague Dawley rats (Charles River Laboratories) weighing 290–320 g were anesthetized with isoflurane (5% for induction, 2% for maintenance) in N_2_O/O_2_ (70:30%) during surgical procedures, and the body temperature was maintained at 37.5±0.5 °C using a heating pad. The rats were randomly divided into 5 groups base on the middle cerebral artery occlusion (MCAO) duration: 0, 1.5, 3, 4.5 and 24 hours of MCAO followed by 5-min reperfusion, as previously described^12^. The number of rats in each group is indicated in figure legends. The rats that displayed neurologic deficit typical of MCAO, circling to the left (non-ischemic side), prior to reperfusion were included in this study. One rat without circling was excluded. Totally, sixty rats were used in this study.

### Measurement of BBB permeability

A subset of 30 rats were used for BBB permeability measurement as described previously^34^. Briefly, 2% Evans blue was injected immediately after removal of the suture for reperfusion. After 5-min reperfusion, the rats were perfused with ice-cold PBS. Hemispheric tissues were homogenized in 1 mL 50% trichloroacetic acid, and the contents of the dye were assessed by measuring the fluorescence intensity using Odyssey^®^ Infrared Imaging System (Li-cor, USA) (emission wavelength of 680 nm). The total Evan’s blue content (ng) in each sample was calculated according to an external standard curve. The difference of dye contents between ischemic and non-ischemic hemispheric tissue reflected the extent of BBB damage.

### Measurement of blood biomarkers

For each rat, blood samples (1 ml) were taken from the left femoral vein at three time points: before MCAO surgery, before reperfusion onset, and after 5-min reperfusion. The serum was separated by centrifuging the blood samples at 3000 g for 10 min at 4 °C before measuring the targeted proteins with commercially available enzyme-linked immunosorbent assay (ELISA) kits (occludin: USCN, China; claudin-5: Biomatik, Canada; MMP-9: R&D Systems, USA): 100 μl for occludin, 50 μl for claudin-5 and 50 μl for MMP-9. The remaining serum was used for immunoprecipitation of occludin protein.

### Immunoprecipitation of occludin protein in blood samples

Serum occludin was immunoprecipitated using Dynabeads Protein A immunoprecipitation kit (Life technologies, USA) according to manufacturer’s instruction. Briefly, 2 μg of occludin antibody (Life technologies) was incubated with Dynabeads to form Dynabeads-Ab complex. Then, 100 μl serum sample was incubated with Dynabeads-Ab complex to pull down occludin protein. The occludin protein was eluted out by incubating the beads with SDS sample buffer for 10 min at 70 °C. Then western blot was performed to assess occludin level using an antibody from Santa Cruz Biotech, USA.

### Isolation of cerebral microvessels

Rats were sacrificed by decapitation at the end of reperfusion. Brains were quickly removed and chilled in ice-cold PBS for 5 min. Brains were sectioned to 2-mm thick coronal slices, 2 mm away from the tip of the frontal lobe, which contained the main infarction area according to our earlier studies^35^. Non-ischemic and ischemic hemispheric tissue was then collected and homogenized in 4 ml ice-cold PBS using a Dounce homogenizer. The homogenate was filtered through a 41-μm nylon mesh (Spectrum, USA). After rinsing three times with 5 ml PBS, the microvessels retained on the mesh were washed off with PBS and pelleted by centrifugation at 4000 *g* for 10 min at 4 °C. The pellets were stored at −80 °C until further analysis.

### Western blot analysis for occludin in cerebral microvessels

The isolated cerebral microvessels were homogenized in 50 μl RIPA buffer and centrifuged at 16,000 g for 15 min at 4 °C, and protein concentrations in supernatants were determined using protein assay reagents (Bio-Rad, USA). After separating the samples of 40 μg protein by electrophoresis, the protein levels were analyzed through western bolt by using occludin (diluted 1:250, Life technologies) or GAPDH (diluted 1:1,000, Santa Cruz) as primary antibody and RDye 800 CW goat anti-rabbit and IRDye 680 goat anti-mouse secondary antibodies (diluted 1:10,000, Li-Cor). At last the immunoblots were photographed using the Odyssey^®^ Infrared Imaging System (Li-Cor) with Molecular Imaging Software V4.0.

### Co-immunostaining of occludin and microvessels

Co-immunostaining was performed on 4% paraformaldehyde fixed 16-μm-thick brain slices to colocalize the expression of occludin (antibody dilution 1:50, Life technologies) and rat endothelial cell antigen-1 (RECA-1, antibody dilution 1:30, Abcam, USA). Images were captured on a fluorescence microscope (Olympus IX71).

### Statistical analysis

Data were presented as mean ± SEM. One-way ANOVA followed by Bonferroni post hoc correction was used to analyze the differences in means from groups. A value of P < 0.05 was considered statistically significant. Pearson’s correlation was used to evaluate the linear relationships.

## Additional Information

**How to cite this article**: Pan, R. *et al*. Blood Occludin Level as a Potential Biomarker for Early Blood Brain Barrier Damage Following Ischemic Stroke. *Sci. Rep.* **7**, 40331; doi: 10.1038/srep40331 (2017).

**Publisher's note:** Springer Nature remains neutral with regard to jurisdictional claims in published maps and institutional affiliations.
